# Moving Mountains: Improving Access to Autologous Stem Cell Transplant for Vulnerable Patient Populations

**DOI:** 10.3390/cancers18121967

**Published:** 2026-06-17

**Authors:** Srinivas Devarakonda, Qiuhong Zhao, Suzanne Keirns, Naresh Bumma, Abdullah M. Khan, Francesca Cottini, Elvira Umyarova, Nowshin Islam, Jesse J. Plascak, Electra D. Paskett, Jessica Krok-Schoen, Nidhi Sharma, Don Benson, Ashley E. Rosko

**Affiliations:** 1 Division of Hematology, James Comprehensive Cancer Center, Department of Internal Medicine, The Ohio State University, Columbus, OH 43210, USA; srinivas.devarakonda@osumc.edu (S.D.); qiuhong.zhao@osumc.edu (Q.Z.); suzanne.keirns@osumc.edu (S.K.); naresh.bumma@osumc.edu (N.B.); abdullah.khan@osumc.edu (A.M.K.); francesca.cottini@osumc.edu (F.C.); elvira.umyarova@osumc.edu (E.U.); islam.217@wright.edu (N.I.); nidhi.sharma@osumc.edu (N.S.); don.benson@osumc.edu (D.B.); 2 Division of Cancer Prevention and Control, Department of Internal Medicine, James Comprehensive Cancer Center, The Ohio State University, Columbus, OH 43210, USA; jesse.plascak@osumc.edu (J.J.P.); electra.paskett@osumc.edu (E.D.P.); 3 Division of Health Sciences, School of Health and Rehabilitation Sciences, College of Medicine, The Ohio State University, Columbus, OH 43210, USA; jessica.schoen@osumc.edu

**Keywords:** multiple myeloma, autologous stem cell transplant, rural, older adult, minority, appalachian

## Abstract

**Background**: Multiple Myeloma (MM) remains the most common indication for Autologous Stem Cell Transplant (ASCT). ASCT use is disproportionately lower in older adults, Black patients with MM, as well as those living in rural areas and of low socioeconomic status (SES). Low utilization of ASCT is linked to poor outcomes. There are numerous barriers to ASCT access and limited interventions to address disparate outcomes in patients with MM. **Methods**: This is an ambispective study conducted at a single institution to identify and mitigate barriers to ASCT utilization. In aim one, we retrospectively evaluated ASCT utilization and overall survival (OS) for MM patients accounting for clinical, geographic (Rural Urban Commuting Area—RUCA), and socioeconomic factors (Yost). In aim two, based on the observations of the retrospective study, we conducted a prospective feasibility study to improve access for vulnerable patients with MM (older adults, Black patients, rural and/or Appalachian residence) using a virtual ASCT consultative model with patient navigation, coordinated with local oncology centers. Poisson regression estimated the relative risk of receiving ASCT. OS was calculated from MM diagnosis to the date of death censoring survivors at last contact and analyzed using Kaplan-Meir. Cox proportional hazard models estimated the hazard ratio for risk of death. The multivariable model was built including significant risk factors from the univariable models to estimate the independent effect of each risk factor. **Results**: Among 1799 patients, median age of diagnosis was 61 (range 17–87), race was self-identified as White (85.6%), Black (13.1%), or other (1.3%) and it was primarily metropolitan (n = 1205, 71.2%) and 1169 (65%) received a transplant. The hazard of death was lower for those who received a transplant (vs. no transplant, HR = 0.63, 95%CI 0.53–0.74, *p* < 0.001), patients identified as Black (vs. White, HR = 0.66, 95%CI 0.51–0.85, *p* = 0.001) and higher SES areas (quartile 2–4 vs. quartile 1, HR = 0.69, 95%CI 0.58–0.83, *p* < 0.001). Prospectively with patient navigation and virtual consultation (n = 35), half of the patients sought virtual consultation, 68% of patients were eligible for transplant, 43% went on to receive ASCT, of which 42% of patients were non-metropolitan and 15% of patients were from an Appalachian county. Using this strategy, no difference was observed in ASCT utilization by age (31% in age > 70 vs. 45% in age 65–70 vs. 78% in age < 65, *p* = 0.10), geographical area (47% in metro vs. 50% in non-metro, *p* = 0.99), or race (46% in White vs. 60% in Black, *p* = 0.66). **Conclusions**: Implementing patient navigation and virtual transplant consultation can effectively enhance access to ASCT among underserved MM populations while reducing the influence of racial and geographic differences.

## Simple Summary

Multiple myeloma is a blood cancer that commonly affects older adults. Many patients do not receive autologous stem cell transplantation, a treatment that can improve survival. Barriers such as older age, socioeconomic disadvantage, geographic distance from transplant centers, and limited access to specialized care may contribute to lower transplant utilization. The objectives of this study were to identify factors associated with transplant use and survival and to evaluate whether a model combining virtual transplant consultation and patient navigation could improve access to transplant for vulnerable populations. In a retrospective analysis of 1799 patients, we found that older adults, Black patients, and those living in lower socioeconomic areas were less likely to undergo transplantation. Patients who received a transplant had longer survival. We then prospectively evaluated a virtual consultation and patient navigation program in 35 patients, including older adults and individuals from rural and Appalachian communities. The program was feasible and allowed patients to access expert transplant evaluation without having to travel to a tertiary care center. These findings suggest that telemedicine and patient navigation can help improve access to stem cell transplantation and other advanced cancer care treatments, thereby promoting more equitable healthcare delivery and improved outcomes for underserved populations.

## 1. Introduction

Multiple myeloma (MM) is a disease of aging adults, and more common among Black men [1]. According to the American Cancer Society, an estimated 36,110 new cases and 12,030 deaths from multiple myeloma were expected in the United States in 2025. SEER data from 2015 to 2021 further demonstrate an incidence rate of approximately 7.1 per 100,000 persons and a 5-year relative survival approaching 61% [2,3]. Patients with MM are typically diagnosed, treated, and managed throughout their treatment course primarily in the oncology community setting. Patients with MM are recommended to see a specialist at a tertiary care center at the time of diagnosis due to the complexity of the disease, the rapidly changing therapeutic landscape, and the need for autologous stem cell transplant (ASCT) and/or cellular therapy. ASCT is significantly underutilized for numerous reasons, including access, age bias, and perceived excessive toxicity, despite being the standard of care for eligible candidates for decades. Additionally, patients with MM have better long-term survival when treated at higher-volume and academic tertiary care centers [4,5,6].

ASCT is considered a standard of care for newly diagnosed eligible patients with MM [7]. In recent years, the approval of novel agents has resulted in a doubling of overall survival for MM patients [8]. However, this improvement in survival is not observed across all demographics and populations. Several studies have shown that MM outcomes are influenced by various socio-economic factors such as race, ethnicity, age, socioeconomic status and geographic location [9,10]. The degree to which these factors collectively influence patient access to ASCT and jointly impact overall survival in MM is not well established.

Proximity and access to tertiary cancer centers correlate with disparities in clinical outcomes for MM patients [11]. Black patients, those living in rural communities, and individuals with lower sociodemographic status are among the least likely to receive ASCT for MM. Older adults (>65 years of age) receive ASCT only 9–18% of the time (Odds Ratio (OR) = 0.35) [12]. Geographic location is an important demographic factor that impacts the receipt of ASCT. Little is known about the Appalachian (geographic region located in the central and southern sections of the Appalachian Mountains of the eastern United States) population with MM, the majority of whom live in rural areas. Differential access to transplant adversely impacts the outcomes of MM in these vulnerable populations.

As such, we conducted a single-institution ambispective study to identify barriers to ASCT utilization among vulnerable populations with MM and to improve their access to ASCT. First, we conducted a retrospective analysis of transplant utilization and OS accounting for clinical, geographic, and sociodemographic factors using an established database. Based on the identified barriers, we piloted a prospective study of a virtual ASCT consultative model, integrating patient navigation, to expand accessibility to our tertiary care center for the selected vulnerable populations, including older adults, Black patients with MM, and those living in rural and Appalachian communities.

## 2. Methods

In aim one, we retrospectively evaluated MM patients enrolled in an observational cohort (N = 1799), and then in aim two, prospectively consented patients in a virtual ASCT consultative model (N = 35). A retrospective analysis was conducted of adult MM patients from the Ohio State University Comprehensive Cancer Center (OSUCCC) from 1992 to 2020. Patients included previously consented to the Buckeye Surveillance, Contact, and Research for Multiple Myeloma and Amyloidosis Protocol (NCT01408225), a registry database for patients with suspected plasma cell dyscrasia pursuing care at OSUCCC; and the Bone Marrow Transplant (BMT) registry, an internal database comprising demographic and transplant-specific data. Area-level socio-economic status (SES) was measured by census tract-level (2008–2017) and using Yost indices (a composite of education-, income-, and occupation-related variables) [13]. There were 1432 (95.5%) and 861 (94.7%) potentially geocodable addresses out of 1500 and 909 participants in the Myeloma registry and BMT datasets, respectively. Nearly all of those (Myeloma registry: n = 1423, 99.4%; BMT: n = 855, 99.3%) were geocoded to a street address, then combined with census tracts from the 2010 US Census Topologically Integrated Geographic Encoding and Referencing (TIGER) file. Census tract-level, 2008–2017 annual Yost indices were gathered from a Surveillance Epidemiology and End Results (SEER) webpage [14,15]. The year-specific Yost linkages were 1-year lagged; 2018 diagnosis dates were assigned 2017 Yost. Those who were diagnosed in 2018 or later were also assigned 2017 Yost values, and those diagnosed in 2009 and earlier were assigned 2008 Yost values. Rural-urban status was estimated with census tract linkages to the 2010 Rural-Urban Commuting Area (RUCA) codes [16]. Yost scores were categorized into quartiles where higher quartiles represented higher SES, and RUCA codes were grouped by metropolitan (Primary RUCA 1–3) vs. non-metropolitan (Primary RUCA 4–10). Appalachian counties in the state of Ohio were coded according to the Appalachian Regional Commission [17]. Poisson regression with robust error variance was conducted to estimate the relative risk of undergoing transplant. This analytic approach provides an unbiased estimate of the relative risk when the outcome is common (greater than 10%) [18]. Overall survival (OS) was calculated from the MM diagnosis to the date of death censoring those alive at the last contact and analyzed using the Kaplan-Meier method. Cox proportional hazard models were used to estimate the hazard ratio for risk of death. The significant variables from the univariable analyses (UVA) were potential confounders and thus were included in the multivariable model (MVA). The RUCA and Yost indices were among the primary interests of this project and were thus forced into the MVA, despite their significance in the UVA. MVA was built using patients from the MM registry, and a complete-case analysis was performed due to the larger sample size and low rates of missing data (6.7%). The proportional hazards assumptions were checked using the plot of log(–log(survival)). Since our patients were diagnosed from 1992 to 2020, given the new treatments approved by the FDA (bortezomib in 2003 and daratumumab in 2015), we categorized treatments into 3 eras (1992–2002, 2003–2014, and 2015–2020).

The prospective study involving the virtual consultation and patient navigation model was developed based on selected disparities, namely age, race and patient area of residence, with the goal of addressing these modifiable barriers to ASCT access. This study was specifically aimed at increasing transplant access to older adults (65 years and older), residents of rural or Appalachian counties, and/or Black patients with MM. We utilized virtual consultation and enhanced care coordination with local treating centers by employing a transplant navigator among care team members. During active enrollment, patient navigation and community oncology clinic check-ins were used to help identify patients for ASCT consultation. All patients with MM referred to OSUCCC-James between 2021 and 2024 were screened for the study. Patient charts were reviewed to determine if they met one or more of the following eligibility criteria: age 65 years or older, a resident of a rural and/or Appalachian Ohio county, and/or identified as Black/African American. Classification of the area of residence of patients as rural or urban was done using RUCA codes. The transplant navigators made initial patient contact if deemed eligible to gauge potential participation in the study and coordinate scheduling needs. Patients and/or providers could elect to see the transplant physician in-person for any reason during the evaluation. Due to insurance limitations for virtual consultation for out-of-state patients, only residents of Ohio were considered for study participation. 

Patients provided consent remotely using the REDCap electronic data capture system. Clinical and demographic information was captured at baseline. Findings of pre-transplant evaluation and details of their transplant course were also collected. At baseline, patients were assessed for Health-Related Quality of Life (HRQoL) (PROMIS-GHS), comprehensive geriatric assessment (GA) (if 65 years or older), and access to electronic/digital devices. HRQoL was longitudinally assessed at the time of transplant and during all subsequent follow-up visits. The mean of HRQoL scales at screening and over time including at the time of transplant as well as day 14, day 30 and day 90 post-transplant was calculated, and the trend of line plots was generated using Excel. For those who received a GA at baseline, the survey was readministered 90–100 days post-transplant. At 30 days post-transplant, financial hardship was assessed using transplant financial toxicity instruments as previously described [19]. Patient characteristics and health status were summarized using the median and range, or frequency and percentage depending on data type, and were compared between patients living in metro vs. non-metro, and Appalachian area vs. non-Appalachian area, and by race using the Mann–Whitney test or Fisher’s exact test. All analyses were conducted using Stata Version 18 and the significance level was set at *p* < 0.05.

## 3. Results

A total of 1799 MM patients were included for the retrospective analysis, of which 1169 (65%) received a transplant. MM patients’ median age at diagnosis was 61 (range: 17–87), race was self-identified as White (85.6%), Black (13.1%), or other (1.3%) and was primarily metropolitan (n = 1205, 71.2%). In a multivariable analysis, patients who were 65 years or older were less likely to undergo a transplant (age 65–70: Incidence Rate Ratio (IRR) = 0.80, 95%CI: 0.72–0.89, *p* < 0.001; age > 70: IRR 0.23, 95%CI: 0.18–0.30, *p* < 0.001). When assessed by race, relative to White patients, Black patients were less likely to receive an MM transplant (IRR = 0.85, 95%CI: 0.74–0.97, *p* = 0.018). RUCA coding showed no significant difference in receiving a transplant between the non-metropolitan vs. metropolitan groupings (IRR = 1.06, 95%CI: 0.97–1.16, *p* = 0.186). Residence in higher SES areas by the Yost index (quartiles 2–4) was associated with a higher rate of ASCT receipt compared to those in lower SES, Yost index quartile 1 (IRR = 1.16, 95%CI: 1.05–1.29, *p* = 0.005) (Table 1).

**Figure 1:**
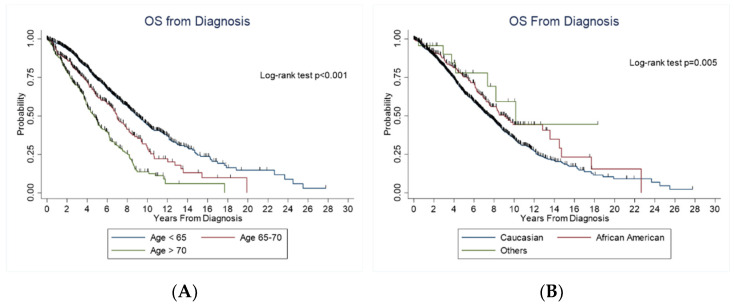
(**A**) Overall survival from diagnosis by age; (**B**) Overall survival from diagnosis by race.

Among all MM patients, the median OS for age < 65 was 9.0 years (95%CI 8.4–9.7); age 65–70, 7.0 years (95%CI 6.3–7.9); age > 70, 4.7 years (95%CI 4.0–5.3). Black patients with MM had a decreased risk of death compared to White patients; the median OS for White patients with MM was 7.5 years (95%CI: 6.9–8.0), Black patients with MM 9.0 years (95%CI: 7.5–13.6), and for others 10.1 years (95%CI: 7.3–NR) (Figure 1A,B). In the multivariable analysis, the hazard of death was lower for those who received a transplant (vs. no transplant, HR = 0.63, 95%CI 0.53–0.74, *p* < 0.001) and for patients identified as Black (vs. White, HR = 0.66, 95%CI 0.51–0.85, *p* = 0.001). Advancing age was associated with a higher hazard of death, age 65–70 HR = 1.45 (95%CI 1.18–1.78, *p* < 0.001); age > 70 HR = 2.03 (95%CI 1.65–2.49, *p* < 0.001). The hazard of death was lower for those residing in higher SES areas by YOST index (quartile 2–4 vs. quartile 1, HR = 0.69, 95%CI 0.58–0.83, *p* < 0.001). Rurality, by RUCA, was not associated with OS (HR = 1.14, 95%CI 0.96–1.35, *p* = 0.134) (Table 2).

**Figure 2:**
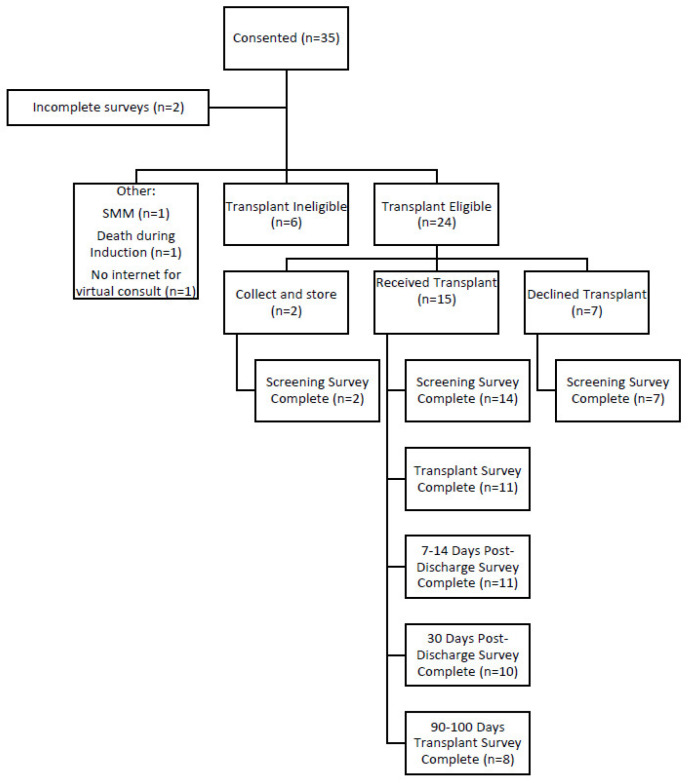
Consort Diagram.

In the prospective aim, 35 patients consented to the study. Pre-ASCT evaluation was done virtually for 18 patients and in-person for 17 patients. Furthermore, 33 patients were included in the baseline analysis (Figure 2). The median age was 68 (range 51–82), 23 (70%) were male, and 5 (15%) patients were Black. Geodemographically, patients were non-metropolitan n = 14 (42%) and 5 (15%) were from an Appalachian area. High-risk MM disease and stage were similar across groups by race, geography (Appalachian), or rurality. In total, 24 (68%) patients were eligible for ASCT, and 6 patients were ineligible. Patients who underwent ASCT were younger (median 65 yo. vs. 73 yo., *p* = 0.02), more likely to be employed (53% vs. 0%, *p* = 0.001), and less likely to be smokers (44% vs. 82%, *p* = 0.027) (Table 3). Financial hardship was present among 63% of ASCT recipients; 30% of those patients reported decreased monthly family household income post-transplant, and 20% of patients reported requiring transplant-related relocation, transportation, or financial changes at home.

**Figure 3:**
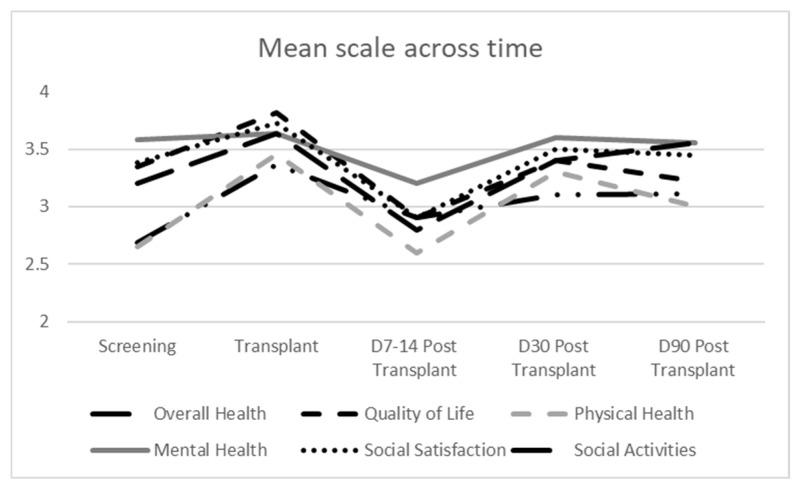
Quality of Life Over Time with ASCT.

Among the entire cohort, Black patients with MM were younger, with a median age of 59 (51–71) compared to their White counterparts with a median age of 68.5 (52–82), *p* = 0.03, but there were no differences in terms of gender, rurality, or disease characteristics such as stage, cytogenetics, treatment, or comorbidities between the two groups (Table 3). There were no differences in QoL among patients by transplant status, geographical area, or race. Social support/activities, marital status, mental health, Instrumental Activities of Daily Living (IADL), performance status, and religiousness or spirituality were also found to be similar among patient groups studied (Supplementary Table S1). We characterized the dynamics of HRQoL at screening and over time including at time of transplant as well as day 14, day 30 and day 90 post-transplant. Across all health measures, majority of patients were in good/very good/excellent condition at screening and at the time of transplantation. A decline was observed at day 14 post-transplant, followed by a return to baseline levels at day 30 (Figure 3).

Compared to non-metropolitan patients, more metropolitan patients a received triplet regimen including bortezomib/lenalidomide/dexamethasone (VRD) (78% vs. 36%, *p* = 0.028) and had higher a self-reported health rating score (90 vs. 75, *p* = 0.007). Patients from Appalachian regions were more likely to be current/former smokers (100% vs. 57%, *p* = 0.025) and were less likely to receive triplet induction regimens (20% vs. 67%, *p* = 0.029). No difference was observed in ASCT utilization in older age (31% in age > 70 vs. 45% in age 65–70 vs. 78% in age < 65, *p* = 0.10), geographical area (47% in metro vs. 50% in non-metro, *p* = 0.99; Appalachian 18% vs. non-Appalachian 13% *p* = 0.99) or race (46% in White vs. 60% in Black, *p* = 0.66) in this virtual consultative model (Table 3).

## 4. Discussion

The frontline landscape of MM treatment continues to evolve. Although ASCT has been the standard of care for decades, the number of patients who receive ASCT across populations is approximately 11% [20]. This is particularly true among older adults, socioeconomically vulnerable populations, and patients residing far from transplant centers. Barriers including travel distance, limited local resources, caregiver dependence, and transportation challenges frequently delay or prevent referral to transplant evaluation. These challenges disproportionately affect older adult patients, who may rely on family members or caregivers for transportation and/or coordination of care.

In our retrospective study, we found that older adults are less likely to receive ASCT, which has been shown in earlier studies, reflecting physical, social, and economic barriers [21]. As we and others have identified, Black patients with MM are least likely to receive an ASCT, yet tend to have improved median OS when provided with similar treatments, including novel therapies and/or ASCT [22,23]. We observed that when examining the geospatial differences, there was no difference in receipt of ASCT or survivability by metropolitan coding, yet those individuals of higher socioeconomic status by Yost index were more likely to receive an ASCT and have improved OS, which may be explained by the interplay of education, income, health literacy, overall health and improved access to novel therapies as outlined by the National Cancer database study by Chamoun et al. [24].

Prospectively, half of the patients sought virtual consultation when given the opportunity to see a transplant physician, of which 42% of patients were non-metropolitan and 15% of patients were from an Appalachian County, confirming the need for dedicated patient navigation in improving access to ASCT for these patient populations. When these patients were provided access via patient navigation and virtual consultation, there was no difference in ASCT utilization by race or geographical location, yet younger patients received ASCT more frequently, likely due to better overall medical and physical health. The small sample size may have reduced the statistical power to detect meaningful differences in the myeloma-related and general health-related characteristics. In addition, deliberate efforts to provide equitable access to Black and rural populations may have helped to reduce the traditional disparities, although the small sample size may have limited the ability to detect statistically significant differences. The drop in the HRQoL immediately following ASCT with subsequent recovery to baseline in our study mirrors the findings in major studies evaluating the role of ASCT in MM [25,26].

In this pilot study, patients residing in rural areas have been shown to have lower utilization of triplet regimens for induction therapy. A similar pattern has been observed even for patients from the Appalachian region. Cancer-related disparities, including incidence and mortality, have been well-documented in the Appalachian regions in the U.S. which are at least partially explained by the socioeconomic and geographical factors [27]. Reduced usage of novel drugs in the treatment of MM could be related to a greater travel distance to academic centers, limited availability of myeloma specialists, lower socioeconomic resources, insurance barriers, reduced access to clinical trials, and infusion infrastructure.

Understanding the challenges and gaps for access to tertiary care is not limited to geographic challenges [28]. According to the 2020 census, approximately 57 million Americans (roughly 20% of the population) live in rural areas [29]. There is increasing evidence for disparities in cancer care among rural populations [30,31,32,33]. When patients have equal access to high-level care, as in the setting of a clinical trial, there are no differences in cancer outcomes among rural and urban populations. This was demonstrated in over 30,000 patients who enrolled in clinical trials across 17 different types of cancer cohorts, including MM, demonstrating no difference in OS, PFS, or cancer-specific survival among patients of rural residence [34]. Delamater et al. reported that half of the patients in the United States can access a transplant center within an hour, and 80% of individuals are within 2 h of geographic distance [35]. However, distance is not the only barrier to ASCT utilization. Socioeconomic differences play a role in both the access to ASCT and its outcomes [23], as we have identified in our analysis where the ASCT receipt and survival differ. By incorporating the Yost index, a composite measure of socioeconomic status, investigators can better capture the multidimensional nature of socioeconomic conditions, beyond single metrics of income alone. This approach helps clinicians identify disadvantaged communities and develop strategies to improve access to specialized care such as ASCT.

Differential access to ASCT impacts the prognosis of MM. In addition to rurality, patients of racial/ethnic minorities and aging adults have disparate outcomes [36,37,38,39]. Older adults [Odds Ratio (OR) 0.35] and Black patients (OR 0.58) are less likely to receive a transplant [40]. Non-Hispanic Black (NHB) patients receive a transplant half as often as non-Hispanic White (NHW) patients [41]. Despite less transplant utilization, Black patients tend to have longer overall survival following an MM diagnosis. Large MM epidemiology studies such as the Veterans Affairs (VA) population [42], MMRF CoMMpass [43], or the SEER registries [44] had discordant results on myeloma survival outcomes by race. Some studies report that NHB patients may have lower prevalence of high-risk cytogenetic disease [45] or greater prevalence of favorable cytogenetic profiles such as the presence of t(11;14) [46]. The explanation for favorable survival, despite fewer transplants, in Black patients with MM is not entirely known. In our data, we report similar favorable outcomes among Black patients with MM. This is consistent with population-based SEER studies which report improved survival of Black MM patients relative to White patients (HR = 0.86, *p* < 0.001) [37].

In the pre- and post-COVID eras, several studies have evaluated the role of telemedicine in delivering acute care with the use of remote monitoring during and after ASCT and CAR-T cell therapy, and facilitating survivorship care following these treatments, demonstrating the safety and feasibility of this approach [47,48,49]. However, studies done to assess the utility of telemedicine and telehealth for pre-transplant evaluation have been mostly conducted in the field of solid organ transplantation [50,51]. Relatively fewer studies have been conducted to evaluate its role in ASCT evaluation [49]. Our study is unique in that it combines the roles of patient navigation and virtual consultation in the pre-ASCT evaluation.

Critical for the implementation of this study was the role of patient navigation and virtual consultation. A patient navigator is a patient-centered health care delivery model to assist individuals in underserved communities to overcome barriers in the cancer care continuum [52]. Transplant care is particularly complex for patients with barriers that are structural, cultural, financial, educational, systematic (un/underinsured) and geographic. Patient navigation overcomes barriers and improves access to care as well as follow-up in underserved populations [53]. The effectiveness of patient navigation has been shown to enhance early detection, diagnosis, treatment, and survivorship outcomes. Studies have shown strong evidence that patient navigation enhances QoL, patient satisfaction, decision-making, and treatment knowledge [54]. In our study, the patient navigator and research team established key partnerships within the community cancer center network to coordinate patient care. This collaboration with key community medical oncology practices allowed our team to provide virtual transplant consultation and perform evaluation of eligible patients to streamline access to high-level care reaching Black patients, non-metropolitan, and Appalachian residents with MM. Transplant and cellular therapy programs with the inclusion and involvement of patient navigators will be best positioned to minimize disparate care for disadvantaged and vulnerable patient populations.

Our study has certain limitations, one of them being a small sample size. Being a pilot project designed for proof-of-concept, the cohort size in the prospective part of the study is small, and selection bias may have played a role in patient engagement. These findings need to be validated in a trial involving a larger population. Single-institution design and restriction to residents of a single state with attendant insurance regulations and payment parity significantly limit the generalizability of our study findings. Also, concerns regarding patient privacy, their confidence in the use of technology and internet connectivity in rural areas should be paid attention to. Nevertheless, this study does prove the feasibility and safety of patient navigation and virtual consultation in improving access to advanced treatment modalities such as ASCT and their role in mitigating the healthcare disparities. We used Yost index, which is a broad area-level validated composite measure incorporating seven key indicators including median house value and rent, to assess socio-economic status of patients in our study. However, the lack of individual socioeconomic measures on the retrospective cohort impacts the accuracy of estimation of neighborhood socio-economic composition and its effect on treatment and outcomes. Given the virtual nature of the initial consultation in the prospective portion of the study, we collected information about functional capacity and HRQoL of the patients at baseline and selected timepoints following ASCT, to ensure that this is a safe method for evaluation for transplant eligibility. However, the study was not designed or powered to perform comprehensive longitudinal analyses of geriatric measures or other patient-reported outcomes assessing HRQoL or financial hardships.

## 5. Conclusions

ASCT access remains a major concern despite its established role in the management of MM, with strong evidence linking disparities in access to inferior outcomes. A model combining patient navigation and virtual consultation represents a promising strategy to expand access to ASCT and make expert assessment more readily available to determine patient eligibility or guide alternative treatment decisions if transplant is considered inappropriate either due to disease or patient-related characteristics. This approach saves patients time and travel while providing expertise and shared decision-making.

Patient navigation for complex diseases like MM allows care coordination, helps overcome logistical and socioeconomic barriers to care, promotes education and advocacy, and connects patients with resources. Complementing these efforts, the rapid expansion of telemedicine has created a timely opportunity to improve access to specialized transplant and cellular therapy consultation by streamlining multidisciplinary care coordination and minimizing the need for repeated long-distance travel. As virtual care becomes increasingly integrated into oncology practice, evaluating telemedicine-enabled approaches to ASCT and other cellular therapy access is both necessary and highly relevant to advancing equitable cancer care delivery.

## Figures and Tables

**Table 1:** Socioeconomic and geospatial analysis of factors impacting Multiple Myeloma ASCT receipt.

Multivariable Analysis *	IRR	95%CI		*p* > z
Race (comparator White)				
Black	0.85	0.74	0.97	0.018
Others	0.69	0.42	1.15	0.154
Age (comparator < 65 y.o.)				
Age: 65–70 y.o.	0.80	0.72	0.89	<0.001
Age: >70 y.o.	0.23	0.18	0.30	<0.001
Socioeconomic Status YOST (comparator Quartile 1)				
Quartile 2–4 (higher SES)	1.16	1.05	1.29	0.005
RUCA (comparator 1–3 Metropolitan)				
4–10 (non-Metropolitan)	1.06	0.97	1.16	0.186

* controlling for cytogenetic abnormalities.

**Table 2:** Overall Survival of Patients with Multiple Myeloma by socioeconomic and geospatial analysis.

Multivariable Analysis *	HR	95%CI		*p* > z
Received a transplant	0.63	0.53	0.74	<0.001
Race (comparator White)				
Black	0.66	0.51	0.85	0.001
Others	0.51	0.24	1.09	0.081
Age (comparator < 65 y.o.)				
Age: 65–70	1.45	1.18	1.78	<0.001
Age: >70	2.03	1.65	2.49	<0.001
Socioeconomic Status YOST (comparator Quartile 1)				
Quartile 2–4 (higher SES)	0.69	0.58	0.83	<0.001
RUCA (comparator 1–3 Metropolitan)				
4–10 (Non-Metropolitan)	1.14	0.96	1.35	0.134

* controlling for cytogenetic abnormalities.

**Table 3:** Patient Characteristics at Enrollment for Virtual Consultation and Patient Navigation.

Characteristics	All (n = 33)	Transplant Not-Received (n = 17)	Received Transplant (n = 16)	*p*	Black (n = 5)	White (n = 28)	*p*	Non-Metro (n = 14)	Metro (n = 19)	*p*	Non-Appalach (n = 28)	Appalach (n = 5)	*p*
Age, median, range	68 (51–82)	73 (52–82)	65 (51–75)	0.02	59 (51–71)	68.5 (52–82)	0.03	65 (52–82)	70 (51–77)	0.4	67 (51–82)	69 (52–74)	0.76
Gender				0.47			0.99			0.71			0.15
Male	23 (70)	13 (77)	10 (63)		4 (80)	19 (68)		9 (64)	14 (74)		21 (75)	2 (40)	
Race				0.99						0.057			0.57
Black	5 (15)	2 (12)	3 (19)		-	-		0 (0)	5 (26)		5 (18)	0 (0)	
White	28 (85)	15 (88)	13 (81)		-	-		14 (100)	14 (74)		23 (82)	5 (100)	
Marital Status				0.99			0.28			0.11			0.28
Divorced/Separated/Single	7 (21)	4 (24)	3 (19)		2 (40)	5 (18)		5 (36)	2 (11)		5 (18)	2 (40)	
Married	26 (79)	13 (77)	13 (81)		3 (60)	23 (82)		9 (64)	17 (89)		23 (82)	3 (60)	
Employment Status				0.001			0.65			0.14			0.23
Unemployed	4 (13)	2 (13)	2 (13)		1 (20)	3 (12)		2 (17)	2 (11)		3 (11)	1 (25)	
Employed	8 (26)	0 (0)	8 (53)		2 (40)	6 (23)		4 (33)	4 (21)		8 (30)	0 (0)	
Retired	17 (55)	12 (75)	5 (33)		2 (40)	15 (58)		4 (33)	13 (68)		15 (56)	2 (50)	
Disabled	2 (6)	2 (13)	0 (0)		0 (0)	2 (8)		2 (17)	0 (0)		1 (4)	1 (25)	
Metropolitan				0.99			0.06						0.14
No	14 (42)	7 (41)	7 (44)		0 (0)	14 (50)		-	-		10 (36)	4 (80)	
Yes	19 (58)	10 (59)	9 (56)		5 (100)	14 (50)		-	-		18 (64)	1 (20)	
Appalachian Area				0.99			0.57			0.14			
No	28 (85)	15 (83)	13 (87)		5 (100)	23 (82)		10 (71)	18 (95)		-	-	
Yes	5 (15)	3 (18)	2 (13)		0 (0)	5 (18)		4 (29)	1 (5)		-	-	
Smoking status				0.027			0.99			0.59			0.025
current smoker	3 (9)	3 (18)	0 (0)		0 (0)	3 (11)		2 (14)	1 (5)		1 (4)	2 (40)	
former smoker	18 (55)	11 (65)	7 (44)		3 (60)	15 (54)		8 (57)	10 (53)		15 (54)	3 (60)	
never smoker	12 (36)	3 (18)	9 (56)		2 (40)	10 (36)		4 (29)	8 (42)		12 (43)	0 (0)	
R-ISS at diagnosis				0.74			0.77			0.99			0.24
1	10 (42)	4 (33)	6 (50)		1 (25)	9 (45)		4 (44)	6 (40)		10 (45)	0 (0)	
2	11 (46)	6 (50)	5 (42)		3 (75)	8 (40)		4 (44)	7 (47)		10 (45)	1 (50)	
3	3 (13)	2 (17)	1 (8)		0 (0)	3 (15)		1 (11)	2 (13)		2 (9)	1 (50)	
Induction regimen				0.99			0.87			0.028			0.029
Carfilzomib/lenalidomide/ dex	1 (3)	1 (6)	0 (0)		0 (0)	1 (4)		1 (7)	0 (0)		0 (0)	1 (20)	
Cytoxan/Bortezomib/dex	1 (3)	0 (0)	1 (6)		0 (0)	1 (4)		1 (7)	0 (0)		1 (4)	0 (0)	
Daratumumab/bortezomib lenalidomide/dex	7 (22)	3 (18)	4 (25)		2 (40)	5 (19)		3 (21)	4 (22)		6 (22)	1 (20)	
Bortezomib Cyclophosphamide/dex	1 (3)	0 (0)	1 (6)		0 (0)	1 (4)		1 (7)	0 (0)		0 (0)	1 (20)	
bortezomib/lenalidomide /dex	19 (59)	10 (63)	9 (58)		3 (60)	16 (59)		5 (36)	14 (78)		18 (67)	1 (20)	
daratumumab/lenalidomide/dex	3 (9)	2 (13)	1 (6)		0 (0)	3 (11)		3 (21)	0 (0)		2 (7)	1 (20)	
Total number of comorbidities, median, range	6 (0–20)	8 (0–20)	4 (1–14)	0.09	5 (3–20)	6 (0–14)	0.91	4.5 (1–13)	6 (0–20)	0.35	6 (0–20)	2 (1–8)	0.21
Total number of concomitant medications, median, range	9 (2–20)	11 (3–20)	9 (2–17)	0.08	11 (9–15)	9 (2–20)	0.21	8.5 (2–20)	10 (3–18)	0.26	9 (2–20)	9 (6–11)	0.64

## Data Availability

The original contributions presented in this study are included in the article/Supplementary Material. Further inquiries can be directed to the corresponding author.

## Supplementary Materials

The following supporting information can be downloaded at: https://www.mdpi.com/article/10.3390/cancers18121967/s1, Table S1: Patient-Reported QoL and Psychosocial Characteristics.
