# Supplementary material for: Moving Mountains: Improving Access to Autologous Stem Cell Transplant for Vulnerable Patient Populations
Source: Cancers (Basel). 2026 Jun 17;18(12):1967. doi: 10.3390/cancers18121967 (PMC13297116; doi:10.3390/cancers18121967)
Supplement: Supplementary file 1 [file cancers-18-01967-s001.zip › cancers-4289728-supplementary.pdf]

Supplement Table S1. Patient-Reported QoL and Psychosocial Characteristics													
**Measures**	**All (n=33)**	**No Transplant (n=18)**	**Received Transplant (n=15)**	**p**	**Black (n=5)**	**White (n=28)**	**p**	**non-Metro (n=14)**	**Metro (n=19)**	**p**	**non-Appalachian (n=28)**	**Appalachian (n=5)**	**p**
**PROMIS score, median, range**	34(22-45)	34(22-39)	34(24-45)	0.77	29(24-38)	35(22-45)	0.32	36.5(22-41)	32(23-45)	0.3	34.5(23-45)	27(22-37)	0.22
**IADL score, median, range**	13.5(8-14)	13.5(8-14)	13.5(11-14)	0.99	13.5(13-14)	13.5(8-14)	0.99	13(8-14)	14(11-14)	0.39	14(11-14)	10.5(8-13)	0.11
**MOS Physical Health Score**	56(17-94)	56(17-94)	61(39-94)	0.53	83(72-94)	56(17-94)	0.13	53(17-67)	69(33-94)	0.076	61(28-94)	36(17-56)	0.23
**Self-Report Health Rating score**	80(60-100)	85(60-100)	80(60-90)	0.5	90(80-100)	80(60-100)	0.46	75(60-80)	90(70-100)	**0.007**	80(60-100)	70(60-80)	0.23
**Comorbidities score**	4.5(2-8)	4.5(2-8)	5(2-7)	0.74	6(5-7)	4(2-8)	0.24	5.5(3-8)	4(2-7)	0.28	4.5(2-7)	5.5(3-8)	0.87
**Mental health score**	88(60-96)	88(60-93)	88(71-96)	0.99	82(71-93)	88(60-96)	0.95	87(60-96)	88(71-93)	0.89	88(71-96)	72(60-83)	0.08
**Social activities score**	50(17-92)	50(17-75)	46(25-92)	0.99	54(42-67)	50(17-92)	0.99	46(17-67)	50(25-92)	0.37	50(25-92)	33(17-50)	0.27
**Social Support Score**	96(67-100)	96(67-100)	96(81-100)	0.87	85(83-88)	97(67-100)	0.26	97(67-100)	96(71-100)	0.76	95(67-100)	100(100-100)	0.29
**Religion/ spirituality score**	18(5-27)	16.5(5-27)	22.5(7-27)	0.31	15.5(15-16)	19(5-27)	0.67	14.5(7-25)	19.5(5-27)	0.44	19(5-27)	14.5(11-18)	0.67
**KPS**				0.57			0.57			0.93			0.84
**70%**	3(11)	2(17)	1(7)		1(25)	2(9)		1(8)	2(13)		3(13)	0(0)	
**80%**	13(48)	5(42)	8(53)		1(25)	12(52)		5(42)	8(53)		10(44)	3(75)	
**90%**	9(33)	5(42)	4(27)		2(50)	7(30)		5(42)	4(27)		8(35)	1(25)	
**100%**	2(7)	0(0)	2(13)		0(0)	2(9)		1(8)	1(7)		2(9)	0(0)	
Instrumental Activities of Daily Living (IADL), Karnofsky Performance Status (KPS)													
